# What Is Shared in an Online Peer‐Support Group Run by Stroke Survivors? An Exploratory Study With a Participatory Approach

**DOI:** 10.1111/hex.70440

**Published:** 2025-09-25

**Authors:** Malin Tistad, Lill Hultman, Jeanette Nelson, Fredrik Sandman, Mikael Åkerlund, Ulla‐Karin Schön, Lena von Koch

**Affiliations:** ^1^ Department of Neurobiology, Care Sciences and Society Karolinska Institutet Stockholm Sweden; ^2^ School of Health and Welfare Dalarna University Falun Sweden; ^3^ School of Social Science Södertörn University Huddinge Sweden; ^4^ Marie Cederschiöld University Collage Stockholm Sweden; ^5^ School of Social Work Stockholm University Stockholm Sweden; ^6^ Theme of Heart and Vascular and Neuro Karolinska University Hospital Stockholm Sweden

**Keywords:** experiential knowledge, Facebook, online peer‐support, participatory, social media, stroke

## Abstract

**Background:**

Many stroke survivors face disabilities that can have a substantial lifelong impact on both their own and their significant others' life situations. Online groups on social media can serve as forums for peer support among people sharing a common experience. However, there is limited knowledge about online peer‐support groups for people living with the consequences of stroke that are initiated and managed by stroke survivors themselves.

**Objective:**

The aim of this study was to explore and describe what stroke survivors and their significant others share in posts and comments in a Facebook group for stroke survivors, initiated and run by stroke survivors.

**Methods:**

In this participatory, exploratory and qualitative study, three stroke survivors, engaged as co‐researchers with no previous research experience, identified Facebook as an important context for sharing experiences and peer support. Data were collected by summarizing posts and comments from a Facebook group over 3 months and analyzed using the principles of inductive qualitative content analysis.

**Results:**

In total, 5 categories and 11 subcategories were created. The categories *Narrating About Everyday Life with the Consequences of Stroke*, *Dealing with the Emotional Struggle of Poststroke Life*, *Making Progress and Enjoying Everyday Life*, *Navigating Societal Services and Societal Demands* and *Continuing Everyday Life* describe narratives, questions and requests for other members' experiences related to challenges and situations that the group's members faced post‐stroke.

**Conclusion:**

We conclude that there is considerable breadth in the topics and issues discussed in the online forum, reflecting that a stroke affects many areas of life for both stroke survivors and their significant others. Furthermore, online peer‐to‐peer support for stroke survivors have the potential to provide a context for sharing experiences and knowledge, and for developing experiential knowledge. Clinical implications are that rehabilitation professionals can inform stroke survivors about the existence of online peer‐to‐peer groups, include the ability to use social media in assessments of activity performance, and, if relevant, support stroke survivors' use of social media.

**Patient or Public Contribution:**

Stroke survivors identified Facebook as a context for the study. They collaborated in data collection, analysis, interpretation and discussion of findings and are co‐authors.

## 1 Introduction

Stroke is a leading cause of death and disability globally [1]. Many survivors face a new reality with lifelong impact on both their own and their significant others' life situations [2, 3, 4]. Visible disabilities may include walking difficulties and changes in physical appearance, while invisible disabilities can encompass, for example, fatigue, communication difficulties and memory problems [2]. Disabilities can be extensive and negatively affect autonomy, social life, relationships, socio‐economic situation and the ability to fulfil meaningful roles [2]. Rehabilitation after stroke is often limited in duration, whereas the processes of finding meaning and adapting to the post‐stroke life may continue several years [3, 5]. Many stroke survivors report feelings of emptiness and abandonment, a lack of information about stroke, its consequences, recovery, secondary prevention and medication, as well as a lack of emotional and social support [6, 7].

Peer support can be defined as a ‘system of giving and receiving help founded on key principles of respect, shared responsibility, and mutual agreement of what is helpful’ [8] and is described as being built on shared experiences and understanding of others' situations. Sharing experiences of being a stroke survivor has the potential to meet affirmational, emotional and informational needs by validating concerns, offering a sense of belonging, and providing relevant knowledge to solve everyday problems [9, 10]. Being with peers has been reported to contribute to feelings of comfort and safety, and a sense of community that cannot be found elsewhere [11, 12, 13]. Peer‐support groups that meet in person, most often led by health professionals working in pairs with peer leaders [9, 10, 14, 15, 16] and occasionally by stroke survivors alone [17], are currently the dominant form of peer support. Less is known about other forms of peer‐support poststroke.

Online peer‐support groups have the potential to provide ongoing support and knowledge following the completion of rehabilitation. Therefore, increasing knowledge about such groups, initiated and run by different stakeholders, is significant. A number of studies have investigated one particular online forum for stroke survivors administered and moderated by a stroke organization in the United Kingdom [18]. Commonly discussed topics in the groups included physical symptoms, underlying causes of stroke and potential for recovery [18]. Further, specific post‐stroke topics discussed by the group that have been thoroughly explored include secondary prevention [19], staying in work [20], emotional adjustments [21] and fatigue [22]. Patient organizations in other countries may not have similar roles and opportunities to provide online platforms for peer support, nor do all stroke survivors wish to engage with such organizations. Hence, it is essential to expand knowledge about online peer support and explore forums that are initiated and managed by other stakeholders. Facebook is a social media platform where people with personal experience of a condition can easily start and run groups to share experiences and support each other. Hence, the purpose of this study was to explore and describe what stroke survivors and their significant others share in posts and comments in a Facebook group for stroke survivors, initiated and run by stroke survivors.

## 2 Methods

This study is a participatory, exploratory and qualitative study [23]. Participatory research emphasizes collaboration with communities and stakeholders, including those with lived experience, who may be involved to varying degrees throughout the research process [24]. Stroke survivors involved in research have so far mostly been engaged in limited parts of the process [25], and there is a demand for studies where they are involved across the research cycle. This study was conducted as part of a research project, ‘Participation in Society After an Acquired Brain Injury’, with the overall purpose of exploring how people with acquired brain injuries (ABI) share experience‐based knowledge in different contexts. The participatory approach entailed recruiting and engaging three stroke survivors (M.Å., J.N., and F.S.) with no previous research experience. They are referred to as ‘co‐researchers’ and were recruited through a combination of purposeful selection—reflecting diverse life situations (see paragraph on reflexivity)—and convenience sampling based on contacts. The co‐researchers were involved in all phases of the research project. They participated in early discussions that shaped the research questions for the grant application and contributed at various levels across the project's sub‐studies. The co‐researchers collaborated with two academic researchers (M.T. and L.H.) in weekly meetings between March 2021 and July 2023, and meetings on average each month between August 2023 and December 2024. The meetings typically lasted 1.5 h, and the co‐researchers were compensated for their participation. The meetings included mini‐lectures and discussions about the research process, and planning and execution of the project's studies. In this study, the co‐researchers played an active role throughout the process including identifying the study context, carrying out data collection, analysis and interpretation, and shaping the discussion section.

### 2.1 Study Context and Procedures

The co‐researchers identified Facebook as an important platform for sharing experience‐based knowledge of living with the consequences of an ABI. They suggested two Facebook groups relevant to the study, as these featured an active and continuous exchange of experiences and knowledge. We contacted the administrators of these groups and informed them about our study. One group's administrator declined to participate and the other requested additional information about the study's purpose, data collection methods and the integrity of the Facebook group's members. After receiving the requested information, the administrator agreed to participate on behalf of the group.

The Facebook group under study was a private group founded in 2018, with just over 2000 members. In an average month, more than 50 individual members published posts, and additional members published comments. The group's stated aim was to involve people who believe in life after stroke, to support stroke survivors in recovering as successfully as possible, and to be a forum where members could help each other, find new friends and engage in meaningful discussions. The group also included individuals connected to stroke survivors, whom we refer to as significant others. The group administrators did not take an active role in moderating posts or discussions but occasionally wrote posts and commented on others' contributions. To the best of our knowledge, there were no non‐profit digital platforms with peer support for stroke survivors available in Swedish at the time of the study.

### 2.2 Data Collection

Data were extracted from the Facebook group over a 3‐month period, from December 2021 to the end of February 2022, by summarizing each post and their accompanying comments published during the first 2 weeks. A 3‐month period was considered sufficient to gather data reflecting the topics discussed. In total, 397 posts were published during this period. To safeguard the members' integrity, we did not download or copy posts; instead, we summarized posts and comments directly from the feed.

A protocol developed for this study guided the data extraction. The protocol, created with input from all co‐authors, specified the following items: (1) the author of the post (stroke survivor/significant other/unknown), (2) the author's sex (man/woman/unknown), (3) who the post was about (stroke survivor/significant other/unknown), (4) the type of post (story/question/photograph/video/sharing of link or invitation/quotation/other), (5) a summary of the post's content and (6) a summary of the comment thread. The summaries were constructed to include the core content of the posts and comment threads. To ensure consistency in data collection, M.T. and L.H. initially, together, carried out data extraction from some Facebook posts and comments using the protocol. Subsequently, the entire co‐research group jointly conducted data extraction during a prolonged weekly meeting, discussing how the posts and comments should be summarized and made clarifications in the protocol when needed. The data were then extracted by M.T. or L.H. together with one of the co‐researchers (M.Å., J.N., F.S.) or by L.H. alone. The co‐researchers' reflections on posts and comments were noted. Additionally, each post was assigned a serial number and a descriptive title.

### 2.3 Ethics Approval

Data were collected in a way that respected the private Facebook group members' integrity. Measures were taken to ensure that the process was transparent to the group's members. Digital information meetings, organized as ‘events’ on Facebook, were held before data collection started and once during the data collection. On each occasion, two meetings were held at different times on the same day to enable participation. Additionally, we created a video with information about the study. During the data collection period, information about the study, along with a link to the video, was posted in the Facebook group.

To ensure that individuals in the Facebook group could not be identified, data on names, places or details of events were not extracted. To further minimize the risk of identification, ‘he/him’ and ‘she/her’ have been replaced with ‘they/them’ in the result. The study was approved by the Swedish Ethical Review Authority (2018/407‐31 and 2021‐03548).

### 2.4 Data Analysis

Inspired by a process for involving lay partners in qualitative data analysis described by Garfield et al. [26], we prepared the co‐researchers and conducted the analysis with the aim to ensure ample opportunities for them to reflect and exert real influence. While Garfield and colleagues used thematic analysis, we applied qualitative content analysis, a method suitable for achieving concise yet rich descriptions of the phenomenon under study [27]. A 1.5‐h training session on open coding was held with the co‐researchers, during which the principles of qualitative content analysis, open coding, and the creation of subcategories and categories were introduced and practiced.

The analysis followed the phases of qualitative content analysis, including preparation, organizing and reporting [27]. In the preparation phase, the summaries of the 397 posts were selected as the units of analysis, henceforth referred to as ‘summaries’.

To gain an overview, M.T. and L.H. worked jointly with one or two co‐researchers to make a preliminary sorting of the summaries based on the descriptive titles assigned during data extraction. In the organizing phase, the co‐researchers, on eight occasions totalling nearly 12 h, worked jointly on open coding of a purposive selection of summaries based on the descriptive titles, and on creating subcategories. In the open coding, each summary was kept together as a whole and was assigned one code. M.T. guided the co‐researchers and encouraged them to lead the process, documented the analysis and the co‐researchers' reflections. These sessions resulted in a matrix of subcategories that M.T. used in the subsequent analysis of the remaining summaries. M.T. had regular meetings with the co‐researchers to discuss the development of further subcategories and the creation of preliminary categories. The matrix with a preliminary set of categories and subcategories was thereafter presented to L.H. The matrix was thoroughly discussed, and it was decided to re‐structure some categories and subcategories. M.T. and the co‐researchers thereafter created a new matrix of 13 categories, which was presented to and discussed with all co‐authors. Based on this discussion, M.T. further merged the categories through iterative discussions with all co‐authors, reaching agreement on the five final categories.

In the final step, the protocols with the summaries of the comment threads were revisited. Each summarized commentary thread was linked to its original summary, synthesized and integrated into its subcategory.

### 2.5 Reflexivity

The research team consisted of three female academic researchers: two health care services researchers (M.T. and L.v.K.) and one researcher within the field of social work (L.H.), all with prior professional experience working with stroke survivors. At the time of the study, they held academic positions as senior professor (L.v.K.) and senior lecturers (M.T. and L.H.). They shared a common perspective on the importance of making experiential knowledge and lived experiences visible, recognizing these as unique and valuable forms of knowledge.

In addition, the team included the co‐researchers: two males (M.Å. and F. S.) and one female (J.N.), aged between 51 and 66, and with 8–17 years of lived experience of stroke, including disabilities such as aphasia and hemiparesis. One co‐researcher had a background in physiotherapy. At the time of their strokes, all co‐researchers were employed, although none worked within the healthcare sector. As a result, they had direct experience with return‐to‐work assessments; however, none had returned to employment. Two co‐researchers had children (F.S. and J.N.), two were living alone at the time of the study (F.S. and M.Å.), and two were involved in patient organizations (M.Å. and F. S.). All co‐researchers were or had been members of Facebook groups, including the one examined in this study.

Throughout the research process, ongoing discussions between academic researchers and co‐researchers, representing their different perspectives and lived experience, enabled the team to critically reflect on and become aware of their preunderstandings.

## 3 Results

The 5 categories and 11 subcategories are displayed in Table 1 and described below. Examples of summaries of posts and comments representing each subcategory are displayed in Table 2. It must be noted that the analysis is based on post summaries. Since the comments may address different topics than the posts, some overlap between subcategories appear, reflecting the dynamics and varied nature of the Facebook group discussions.

**Table 1:** Categories and subcategories.

Categories (number of posts)	Subcategories (number of posts, stroke survivors/significant others)
Narrating About Everyday Life with the Consequences of Stroke (*n* = 110)	Living with Stroke and Handling Everyday Tasks (*n* = 71/16)
	Training as Part of Everyday Life (*n* = 22/1)
Dealing with the Emotional Struggle of Poststroke Life (*n* = 38)	The Emotional Burden of Setbacks (*n* = 19/3)
	A Heavy and Frustrating Life Situation (*n* = 12/4)
Making Progress and Enjoying Everyday Life (*n* = 46)	Attaining and Enjoying Achievements (*n* = 29/1)
	Living a Good Life and Appreciating Support (*n* = 14/2)
Navigating Societal Services and Societal Demands (*n* = 67)	The Path to or from Work (*n* = 21/0)
	Interacting with Health Care Services (*n* = 43/3)
Continuing Everyday Life (*n* = 136)	Sharing Recipes and Pictures of Food (*n* = 21/0)
	Social and Leisure Activities (*n* = 64/2)
	Greetings and Words of Wisdom (*n* = 48/1)

**Table 2:** Examples of summaries of posts and examples of comments in comment threads for each subcategory.

Subcategories	Examples of summaries of post with pictures and videos	Examples of comments in comment threads
Living with Stroke and Handling Everyday Tasks	Introduce himself/herself^a^ as a new group member who had a stroke last week that resulted in loss of visual field. He/she didn't think it was a stroke, but partner urged him/her to go to the hospital. At the local emergency room, he/she was sent by ambulance to the larger hospitals, was X‐rayed and told that it was a stroke. Doctors predicted that he/she would get most of vision back. Now, a week has passed, he/she is starting to get scared that it won't come back.	Comment that visual field loss can be reversed and that driving licences can only be temporarily revoked. Vision can return within 3 months, even up to 6 months after a stroke. Loss of visual field in one eye increases the chance of keeping a driving license. Double vision can be reversed. One's eye surgery has been delayed due to COVID.
Training as Part of Everyday Life	Started doing needlework to train eye–hand coordination. *Photograph of work in progress depicting flowers created by pasting beads*.	Some comment that they want to try doing needlework themselves or that it could be an opportunity for relatives who have suffered a stroke.
The Emotional Burden of Setbacks	Gets angry about his/her fumble. Has dropped a garbage bag so that all the contents ended up on the floor. *Photograph showing the empty bag and the food scraps on the floor*.	Some comments about being tired of living with the consequences of stroke and wishes to have life back as it was before. One person recognizes himself/herself in the description and writes that his/her fumbling occurs in periods. Several individuals provide examples of things they have spilled; coffee, sour cream, porridge.
A Heavy and Frustrating Life Situation	His/her partner ignores everything and does not want to take medication, exercise or use aids. The author is completely exhausted.	Suggestions to make a trip, so that the partner understands that he/she needs to exercise to be able to do fun things. There will be periods when it feels difficult to train and that it is easy to lose abilities without training. One needs to give time, show care and love. They as a couple may need support from a counsellor or psychologist.
Attaining and Enjoying Achievements	*Video of woman walking around in grocery store without aids*.	She is good. It is cool. It looks very good. Some individuals wish they could also walk without support.
Living a Good Life and Appreciating Support	Grateful to be able to get support from horse, dog and baby, on the way back from stroke. *Photographs showing a horse, a dog and a baby*	Great with support. Question about how he/she is doing today and tells that his/her husband recently had a stroke that resulted in fatigue. The author replies that he/she is almost physically recovered and provides tips on doing balance exercises and avoiding stress.
The Path to or from Work	Reconciliation meeting with the social insurance office and employers about continued job training at 25%. Disappointed about not having been offered part‐time job by his/her employer because he/she perceived that it worked well with assigned tasks.	Encouragement to contact a union. The next step is to get up to 50% job training. It can be tough. He/she should get help because the employer has an obligation to try to find suitable tasks before concluding that dismissal is the only solution. The author replies that he/she is not a member of a union but will check it out.
Interacting with Health Care Services	Ask how often others have follow‐up visits in healthcare. Have been informed that he/she should have it once every year and feel that it is too rare.	Some individuals have no planned follow‐ups and contact the healthcare themselves if necessary. One person with several different healthcare contacts due to other diagnoses experiences that no healthcare provider takes responsibility but refers to someone else. Some individuals have annual follow‐up visits via rehabilitation services or in primary care.
Sharing Recipes and Pictures of Food	He/she made own tortillas. *Photograph of a baking sheet with tortillas*.	Several comments that it looks good and that it's well done.
Social and Leisure Activities	That he/she has spent time with his/her daughter and grandchildren. *Photograph of people ice‐skating and scootering*.	It looks lovely.
Greetings and Words of Wisdom	Never forget how far you've come and everything you've been through. All the times you've fought on, even if it felt like it wasn't going to work. Every morning you've gotten out of bed, no matter how bad you've felt. All the moments you wanted to give up but still managed a new day.	The text is a good inspiration. One should fight on and not give up. One should give oneself credit for what one is doing. Thanks for the reminder.

*Note:* Descriptions of photographs and videos are in italics.

a The expressions he/she, him/her, herself/himself and ‘the author’ are used as deemed most appropriate to facilitate readability while respecting the integrity of the members of the Facebook group.

Most posts were written by stroke survivors (*n* = 364). When referring to any of the 33 posts written by a significant other, it is explicitly stated.

### 3.1 Narrating About Everyday Life With the Consequences of Stroke

Posts in this category described experiences of everyday life with impairments and disabilities post‐stroke, the impact on daily life, and how everyday challenges could be managed and improved. The intention appeared to be to describe and share daily life without focusing on the potential emotional aspects.

*Living with Stroke and Handling Everyday Tasks* described everyday life with the consequences of stroke, along with wishes to share others' knowledge and experiences. The narratives focused on depicting how life was or how problems and challenges could be resolved and managed, to state the circumstances without complaining or describing any mental or emotional impact.

There were chronological narratives from the onset of the stroke to the present day, detailing improvements made and remaining disabilities. These posts sometimes served as introductions for new members or for members to commemorate their ‘survival day’, that is, the anniversary of their stroke incident. Some posts conveyed a sense of being a fighter and having overcome the stroke, along with calls to continue the fight. In the comments, authors were congratulated, their roles as fighters or survivors were affirmed, and the possibility of living a good life despite disabilities was emphasized.

Other posts described the current situation, such as a diminished capacity to reason, challenges in using the subway, or avoidance of social gatherings. They could also include specific events, like visiting a bathhouse and being unable to find one's locker in the changing room or engaging in too many activities and experiencing severe fatigue. Authors often received requests for more details in the comments and were thanked for sharing, especially when slightly humorous stories were conveyed.

Members also wanted to share in others' experiences and knowledge related to various stroke‐related problems and practical challenges of everyday life, including sleeping problems, recovery from fatigue or paresis, as well as more specific questions about mobile phones that could withstand being dropped, affordable support for ice‐skating, or how to manage with one hemiplegic side and the other in a plaster cast. In the comments, members provided concrete suggestions involving strategies, including advice about assistive devices and exercises thought to improve functioning. Additionally, members contributed time perspectives, such as how their fatigue was experienced and managed now—12 years post‐stroke—, or what it was like to be unable to move one's hand 6 years post‐stroke without giving up. Misunderstandings sometimes occurred. For example, questions about how people with only one hand did their nails were initially understood as being about how to paint nails and were deemed non‐serious. After some clarifying questions, the true meaning—how to cut nails—became clear, and many tips were shared on both aids, such as nail scissors with suction cups, and requests for assistance.

Occasionally, significant others, often partners, raised questions and sought to share the members' knowledge and experiences. Significant others who asked questions about how to support their loved ones with stroke often received engaging advice in the comments. However, it seemed important to maintain an accepting and supportive attitude towards the person with stroke. One example involved partners who were trying to encourage their spouses with stroke to do something and wondered how others handled such situations. These partners were initially questioned because their posts were perceived as condescending towards the spouses with stroke. After explaining that the spouses had aphasia, said ‘no’ to every suggestion, and seemed depressed, many members contributed their knowledge and experiences regarding communication difficulties, as well as understanding of the situation from the perspective of a significant other.

*Training as Part of Everyday Life* captured posts about the training members did in daily life to improve functioning or stay fit. Members described practicing activities such as needlework, special painting techniques, or using a toolkit with a knife, scissors and axe to train fine motor skills. Others portrayed more traditional training in gyms and how they adapted exercises to their impairments. With very few exceptions, these posts included photographs or video clips showing exercises or equipment. The posts received encouraging comments, with other members describing their everyday training, cheering, and affirming that exercise is beneficial but requires time and perseverance. One comment thread focused on the physical sensation of regaining function in one's hand, with several members confirming the significance of this and emphasizing how amazing it is when it happens.

### 3.2 Dealing With the Emotional Struggle of Poststroke Life

The posts in this category differed from those in the previous category by emphasizing the emotional impact rather than the events themselves. They reflected that life post‐stroke could involve short‐term emotional impacts caused by setbacks, but also that feelings such as grief, anxiety and frustration sometimes characterized the entire or large parts of the life situation.

*The Emotional Burden of Setbacks* described experiences of fear, anger, worry and grief due to everyday events. Examples included photos of garbage dropped on the floor, accompanied by text expressing anger and frustration at the clumsiness following a stroke. These posts generated many comments that validated the authors' feelings and expressed a longing for life pre‐stroke, depicting life post‐stroke as a struggle. Other posts expressed grief over exclusion from important contexts, such as work, or not being invited to social events. These posts generated many comments reflecting indignation that the authors had not been treated correctly and should not accept the situation and how they had been treated.

Feelings of worry concerning health issues, such as the fear of having to amputate a foot, intertwined with the feeling of having received poor‐quality care, were often expressed. Fear of having another stroke was described in some posts which often generated comments that validated the author's feelings by describing similar experiences. Posts indicating low‐quality care were met with comments urging members to continue searching for better care and to fight by complaining and being persistent.

*A Heavy and Frustrating Life Situation* expressed hopelessness, worries, grief or exhaustion over aspects of, or the entire, life situation. Hopelessness was expressed regarding unfulfilled hopes that life would improve and the endless struggle with strenuous everyday activities. These posts generated comments that validated the authors' experiences by sharing similar situations, such as being isolated due to difficulties in getting around outside with a wheelchair or being a parent responsible for a toddler and household while struggling with everyday activities.

Some posts were written by significant others who expressed exhaustion from caring for a spouse or parent with a pronounced disability after a stroke. Some of these posts seemed to be perceived as lacking sufficient acceptance and understanding towards the stroke survivors, leading to the significant others' actions and experiences being questioned. For instance, temporarily accommodating one's spouse in a nursing home resulted in accusations of giving up too easily but also understanding comments that the burden of care can be unreasonably high and that societal support for spouses is inadequate.

### 3.3 Making Progress and Enjoying Everyday Life

The posts in this category reflected emotional reactions of satisfaction and thankfulness for the life situation, but also a quest for improvement. They also depicted the possibility of regaining the ability to perform activities many years post‐stroke.

*Attaining and Enjoying Achievements* described successes in managing activities and specific situations. A common achievement was related to walking, such as managing to walk in the grocery store, being able to walk longer distances or walking one's child to school. Some posts provided timestamps showing that progress was possible long after the stroke incident, such as making the bed for the first time in 20 years. The posts often included descriptions of hard training and a video or photo showcasing the achievement. The comments contained encouraging cheers, congratulations on the progress, questions about the training and exhortations to take it easy. Members compared their levels of functioning, expressed wishes to be able to walk independently and discussed whether it takes too much energy to both work and make the bed. Other achievements included regaining a driver's license or managing to watch a hockey game without getting tired. Some members wrote that they wanted to give others hope by sharing their progress, while comments depicted experiences of doctors being too cautious about giving hope.

*Living a Good Life and Appreciating Support* reflected a sense of contentment with current life situations and gratitude for support from people in the surrounding environment. A few posts written by significant others described how they experienced an improved life situation and less worry because their loved ones with stroke had resumed a more active life with their own social activities. Thankfulness was directed to friends, family members, pets and the Facebook group.

### 3.4 Navigating Societal Services and Societal Demands

This category described situations where individuals struggled to manage the consequences of their stroke while dealing with society's demands and regulations for returning to work, as well as their needs for healthcare services.

*The Path to or from Work* depicted a trajectory from sick leave after a stroke to an uncertain end goal. The trajectory towards work included unwanted messages about being expected to work full‐time, not being offered desired part‐time work, and hopes of gaining access to vocational training. Members' questions about returning to work reflected processes experienced as sometimes unpredictable and complex, involving several actors. These included how assessments of working capacity were done, how quickly others had returned to work, what happened after vocational training, and access to support. The comments often provided guidance and concrete suggestions, including to contact healthcare, the trade union, and the job centre for support, as well as to prepare thoroughly for meetings by writing down questions. A gradual return to work was recommended by many, and some comments discussed whether working full‐time was worth the risk of health setbacks. Other topics in the comments included the value of work from an economic perspective and as a source of personal meaning and self‐worth but also that life should consist of more than just working, eating and sleeping. Other posts described a direction towards sickness compensation and the joy of being notified that it had been granted. Members who had been granted sickness compensation were congratulated, but it was also acknowledged that sickness compensation could entail a sense of not being good enough.

*Interacting with Health Care Services* described experiences of healthcare contacts across various settings. A few posts detailed ongoing events, such as visits to the emergency room prompted by findings on a check‐up X‐ray. In later updates, members narrated that they had had another stroke without realizing it. These posts generated over 100 comments, many of which discussed stress as a reason for the recurrent stroke and included requests to take it easy and rest. Other posts depicted recent experiences of healthcare encounters, ranging from life‐threatening events like heart attacks to everyday reports from sessions with physiotherapists. In the comments, members shared similar experiences of heart problems or asked questions about physiotherapy sessions and recovery. On several occasions, discussions in the comments drifted from the experiences of healthcare encounters to related topics, unveiling differences between healthcare regions in Sweden in terms of access to rehabilitation and fees for assistive technology.

Many members wanted to share other's experiences with rehabilitation services, such as which services they had received for specific conditions like visual or hearing impairments caused by stroke and stroke without hemiplegia, how often they had follow‐up visits, and how long it was reasonable to wait for an appointment with a speech and language therapist. Some of these posts generated long and rich discussions in the comments that sometimes did not answer the questions but covered experiences of not having had access to the requested rehabilitation for various reasons, including feelings of age‐related discrimination in access or denial of access due to COVID‐19. Both satisfaction with rehabilitation and distrust of healthcare were depicted. Distrust manifested itself in comments emphasizing the importance of only trusting oneself and taking full responsibility for one's own situation.

Posts about a driving ban post‐stroke often generated a significant number of comments depicting variations in how this was handled by physicians. Both 3‐ and 6‐month driving bans were described as standard, but there were also instances of no driving ban at all, in mutual as well as written agreements with physicians.

### 3.5 Continuing Everyday Life

This category described posts that did not mention the stroke or its consequences. The posts reflected a continuation of everyday life, including social and leisure activities, cooking and celebrating holidays. However, the constant presence of the stroke and its impact was depicted in the comments.

*Recipes and Pictures of Food* contained, almost without exception, photographs. The continuation of everyday life was reflected by the focus on food, recipes and weekly menus shared to inspire others. The posts often included a short description of the dishes that had been prepared and generated short appreciative comments, with recipes being requested. The comments portrayed other members' wishes to resume more cooking and baking. On a few occasions, the posts about food sparked discussions about the appropriateness of posting pictures of food when members could be on a diet, and the nutritional content of some dishes was questioned. The critical comments did not receive much support, however, and most members who commented appreciated the food and recipes. It was also argued that all members were adults and could take responsibility for their own eating habits.

The largest single subcategory, *Social and Leisure Activities*, described members' day‐to‐day life. Common topics, often illustrated with pictures, included activities with family and grandchildren, walks and pets. The comments mostly confirmed the joy of these activities but sometimes also expressed fear of doing specific activities after the stroke or a desire to resume a leisure activity. Even though the posts did not mention the stroke, there were sometimes indirect references to it, such as being elected to the board of the local Stroke Club.

*Greetings and Words of Wisdom* included posts with wishes for a Happy New Year and Happy Holidays, as well as the welcoming of new members to the group. The former generated short greetings, while the latter could lead to longer threads of comments where new members introduced themselves and shared their stroke stories. Long‐time members welcomed the newcomers to a supportive group, described the group as an important place to share joy and sorrow, and offered physical meetings if someone lived nearby. The words of wisdom focused strongly on continuing to struggle, taking responsibility for how one's life turns out and finding balance and enjoyment in life. Comments on these posts often reinforced the message, whether it was about keeping up the fight or finding balance.

## 4 Discussion

In this study, we aimed to increase knowledge about online peer support groups for stroke survivors by exploring what stroke survivors and their significant others shared in posts and comments in a Facebook group for stroke survivors, initiated and administered by stroke survivors. Members of the Facebook group appeared to value sharing experiences of everyday life with stroke, including problem‐solving, emotional struggles, achievements, social activities and family life. In the following sections, we will discuss the value and potential role of sharing knowledge and experiences, the social role of online peer groups, and online communities as a context for developing experiential knowledge.

The value of sharing in others' experiences of various aspects of life post‐stroke and handling the consequences of stroke permeated the results but was particularly prominent in the categories *Narrating About Everyday Life with the Consequences of Stroke* and *Navigating Societal Services and Societal Demands*. Fact‐based questions were raised, but most often, members wanted to know how other stroke survivors or significant others had experienced their trajectories through the welfare system, the consequences on everyday life, and how they solved everyday problems. Similar findings have also been reported from an online peer‐support group run by a stroke association where members shared experiences and supported each other across various aspects of life after stroke [18, 19, 20, 21, 22]. The importance of sharing these experiences aligns with findings about the value of participating in in‐person peer groups and sharing experiences that are relatable to one's life situation, allowing for social comparison [10, 12, 28, 29], fostering a sense of belonging, and contributing knowledge and problem‐solving strategies [10, 30]. The desire to share in others' experiences can also reflect the significant responsibility stroke survivors and their significant others often face in navigating a complex and fragmented health and welfare system, where others' experiences might serve as guidance. In this study, the sharing and responses to the sharing seemed to meet the needs of having one's experiences confirmed and assisting peers in managing everyday life, as well as in rehabilitation and return‐to‐work processes. However, there were also misunderstandings in communication, and it must be noted that people with brain injuries have reported concerns with communication when using Facebook [31]. Still, the continuous engagement in the Facebook group in the present study indicates that online communities constitute meaningful and rewarding peer support for those engaging in them.

Posts categorized as *Social and Leisure Activities* were the second most common type, despite not explicitly mentioning stroke. The abundance of posts in this category suggests that the Facebook group served not only as a platform for sharing stroke experiences but also fulfilled a social purpose. The safe environment created among individuals sharing post‐stroke life experiences in face‐to‐face meetings has been described as a community of ‘us’ [12], which is vital for collective belonging and crucial for trying new things without feeling self‐conscious. The Facebook group might have been perceived as a similarly safe environment. Moreover, many stroke survivors experience a shrinking social network, with decreasing contact with friends and fewer opportunities for social interaction due to, for example, fatigue or difficulties participating in activities and conversing with others [3, 32, 33]. In contrast, the Facebook group in this study appeared to provide a context that could adequately respond to anecdotal stories from members' everyday events, making it socially meaningful. Additionally, ‘old’ members sometimes suggested in‐person meetings when welcoming ‘new’ members. Consequently, online peer‐groups for stroke survivors might have the potential to serve dual purposes: providing a safe space for social interaction among peers and offering opportunities to make new friends for in‐person meetings.

The analysis reflects that post‐stroke life may involve prolonged processes of hope, resignation and continuous adjustment. The category *Dealing with the Emotional Struggle of Post‐Stroke Life* highlights that living with the consequences of stroke can involve feelings of hopelessness, setbacks, fear of recurrent strokes and frustration for both stroke survivors and their loved ones. Conversely, the category *Making Progress and Enjoying Everyday Life* reflects progress and the ability to resume activities even 20 years post‐stroke. Aging itself involves changing contextual and personal conditions, and the consequences of stroke have been described as essentially integrated into the normal processes of aging 15 years post‐stroke, rather than being the main concern, although hope for improvements may persist [3, 34]. Findings from this study confirm the enduring hope for improvement and suggest that sharing experiences in an online community can be valuable even decades post‐stroke. Few studies describe these very long‐term perspectives post‐stroke, but a continuous process of reconstructing the self, along with promoting well‐being through attempts to maintain or find new leisure activities throughout the aging process, has been reported [34]. Access to social and supportive networks is crucial in this process [35], which may place stroke survivors in particularly vulnerable positions in the long term if dependent on aging spouses with decreasing ability to provide support [34, 35]. Online peer support might have the potential to offer social support in such processes, and the role of such support should be explored in future studies alongside the potential risks of digital divide and the spread of misinformation that online peer‐support environments might entail.

Generally, posts in the Facebook group indicate that members, based on their illness trajectories, had developed knowledge that could be shared and was useful for peers, referred to as ‘experiential knowledge’ in the literature [36]. Experiential knowledge can be seen as interpretive and pragmatic skills intended for the best possible life with an illness or disability [36, 37]. Such knowledge is developed in interaction with peers through collective sharing of personal reflections in response to specific problems or situations in particular contexts [36], and is hence situated, mirroring the conditions and social identities of the knowledge producers [38]. Research on the development of experiential knowledge has traditionally focused on peer‐support groups meeting in real life [36]. Our findings support previous findings that online peer‐support groups for stroke survivors have the potential to constitute contexts in which experiential knowledge can be developed [13].

The participatory approach was important in this study and our close collaboration over time fostered a sense of safety that encouraged open sharing and valued everyone's experiences and knowledge. The academic researchers strove to ensure that the co‐researchers were involved as much and as actively as they wished, and that they had genuine influence over the process. That academic researchers have more influence and control over the research process must be regarded as reasonable, given their greater expertise and responsibility towards funders. The co‐researchers did not participate in all data coding as they felt it would require more time than they were willing to commit. However, the subsequent analysis process involved iterative and in‐depth discussions with all co‐authors. Overall, we believe that this collaboration enhanced the academic researchers' sensitivity to life post‐stroke and fostered a reflexive approach that enriched the study.

The findings of the study are based on posts published in a single, deliberately selected Facebook group over a 3‐month period. Extending the data extraction period might have revealed seasonal variation in the topics discussed and broadened the findings. However, by the end of this 3‐month period, no new topics came up. Some topics, such as intimacy and sexuality, were completely absent. This absence can be considered unexpected given the importance of these topics and the fact that health professionals rarely address intimacy and sexuality post‐stroke [39]. On the other hand, stroke survivors and their partners might also struggle to discuss these topics [39], and bringing them up in a Facebook group, especially if they are beyond the group's everyday topics, might be sensitive and require courage. In groups that are actively moderated, skilled moderators may have the potential to facilitate discussions about sensitive topics known to be important. Nevertheless, if the group's conversations had been followed over a longer period, or had additional groups been included, these topics, as well as others, might have been covered.

## 5 Clinical Implications

We suggest that rehabilitation professionals can inform stroke survivors and significant others about the existence of online peer‐to‐peer groups, and if relevant include the ability to use social media in assessments of activity performance and support their use of social media. Additionally, the need for support in social media use among stroke survivors with aphasia and cognitive impairment should be acknowledged.

## 6 Conclusion

We conclude that there is considerable breadth in the topics and issues discussed in the online forum, reflecting that a stroke affects many areas of life for both stroke survivors and significant others. Furthermore, online peer‐to‐peer support groups for stroke survivors have the potential to provide a context for sharing experiences, support and knowledge, and for developing experiential knowledge.

## Author Contributions

**Malin Tistad:** conceptualization, data collection, analysis (lead), original draft (lead) review, editing. **Lill Hultman:** conceptualization, data collection, analysis, review, editing, funding acquisition. **Jeanette Nelson:** data collection, analysis, review, funding acquisition. **Fredrik Sandman:** data collection, analysis, review. **Mikael Åkerlund:** data collection, analysis, review, funding acquisition. **Ulla‐Karin Schön:** conceptualization, review, funding acquisition. **Lena von Koch:** conceptualization, review, editing, funding acquisition.

## Ethics Statement

The study was approved by the Swedish Ethical Review Authority (2018/407‐31 and 2021‐03548).

## Consent

Data were collected from a Facebook group and no individual patient/participant consent statement was obtained. We took a series of measures to both inform the members of the Facebook group and protect their privacy, which is described in detail in the manuscript.

## Conflicts of Interest

The authors declare no conflicts of interest.

## Data Availability

The data that support the findings of this study are available on request from the corresponding author. The data are not publicly available due to privacy or ethical restrictions.
